# Association of the *FCN2* Gene Promoter Region Polymorphisms with Very Low Birthweight in Preterm Neonates

**DOI:** 10.3390/ijms232315336

**Published:** 2022-12-05

**Authors:** Agnieszka Szala-Poździej, Anna S. Świerzko, Gabriela Gajek, Maja Kufelnicka-Babout, Karolina Chojnacka, Paulina Kobiela, Dariusz Jarych, Katarzyna Sobczuk, Jan Mazela, Iwona Domżalska-Popadiuk, Jarosław Kalinka, Hideharu Sekine, Misao Matsushita, Maciej Cedzyński

**Affiliations:** 1 Laboratory of Immunobiology of Infections, Institute of Medical Biology, Polish Academy of Sciences, Lodowa 106, 93-232 Łódź, Poland; 2 Department of Perinatology, First Chair of Gynaecology and Obstetrics, Medical University of Łódź, Wileńska 37, 94-029 Łódź, Poland; 3 II Department of Neonatology, Poznań University of Medical Sciences, Polna 33, 60-535 Poznań, Poland; 4 Department of Neonatology, Medical University of Gdańsk, Smoluchowskiego 17, 80-214 Gdańsk, Poland; 5 Department of Neonatology, Poznań University of Medical Sciences, Polna 33, 60-535 Poznań, Poland; 6 Department of Immunology, Fukushima Medical University School of Medicine, 1 Hikariga-oka, Fukushima City 960-1295, Japan; 7 Department of Applied Biochemistry, Tokai University, 4-1-1 Kitakaname, Hiratsuka 259-129, Kanagawa, Japan

**Keywords:** ficolin-2, *FCN2*, newborn, neonate, prematurity, single nucleotide polymorphism, very low birthweight

## Abstract

Single nucleotide polymorphisms (SNPs) localised to the promoter region of the *FCN2* gene are known to influence the concentration of ficolin-2 in human serum and therefore potentially have clinical associations. We investigated the relationships between SNPs at positions −986 (A > G), −602 (G > A), −64 (A > C) and −4 (A > G) and clinical complications in 501 preterms. Major alleles at positions −986 and −64 and A/A homozygosity for both polymorphisms were less frequent among babies with very low birthweight (VLBW, ≤1500 g) compared with the reference group (OR = 0.24, *p* = 0.0029; and OR = 0.49, *p* = 0.024, respectively for A/A genotypes). A lower frequency of G/G homozygosity at position −4 was associated with gestational age <33 weeks and VLBW (OR = 0.38, *p* = 0.047; and OR = 0.07, *p* = 0.0034, respectively). The AGAG haplotype was protective for VLBW (OR = 0.6, *p* = 0.0369), whilst the GGCA haplotype had the opposite effect (OR = 2.95, *p* = 0.0249). The latter association was independent of gestational age. The AGAG/GGAA diplotype favoured both shorter gestational age and VLBW (OR = 1.82, *p* = 0.0234 and OR = 1.95, *p* = 0.0434, respectively). In contrast, AGAG homozygosity was protective for lower body mass (OR = 0.09, *p* = 0.0155). Our data demonstrate that some *FCN2* variants associated with relatively low ficolin-2 increase the risk of VLBW and suggest that ficolin-2 is an important factor for fetal development/intrauterine growth.

## 1. Introduction

The single nucleotide polymorphisms (SNPs) localised to the promoter region of the *FCN2* gene are known to influence the concentration of ficolin-2 (or L-ficolin) in human serum and therefore are considered to have clinical associations. Possession of variant alleles at positions −986 (rs3124952, A > G) and −64 (rs7865453, A > C) is related to lower ficolin-2 levels, while minor alleles at positions −602 (rs3124953, G > A) and −4 (rs17514136, A > G) have the opposite effect [1,2,3]. The above-mentioned SNPs were shown to form 2 haplotype blocks: one created by rs3124952 and rs3124953, and another, by rs7865453 and rs17514136 [4].

Like other ficolins (ficolin-1, ficolin-3) and some collectins [mannose-binding lectin (MBL), collectin-10 (CL-10), collectin-11 (CL-11)], ficolin-2 recognises pathogen-associated molecular patterns (PAMP) exposed on a variety of microorganisms or viruses that enable opsonisation and, by forming complexes with MBL-associated serine proteases (MASP), activation of the complement cascade via the lectin pathway (reviewed in [5,6,7]).

In 2021, in Poland, 24,523 (7.4%) babies were born prematurely, including 1369 delivered before completing 28 weeks of gestation, 2376 at gestational age 28–31 weeks and 20,778 aged 32–36 weeks [8]. Preterm newborns, especially those born at gestational age < 33 weeks and with very low birthweight (≤1500 g), are prone to severe adverse effects, including respiratory distress syndrome (RDS) and perinatal infections, due to immaturity of organs, immune system and other congenital defects. Disorders related to short gestation and low birthweight are the most common causes of neonatal death [9]. Furthermore, very low birthweight (≤1500 g) is considered to continue to influence health during childhood, adolescence and adulthood. It was reported to be associated, for example, with higher risk of coronary heart disease, chronic kidney disease, type 2 diabetes, stroke and hypertension (reviewed in [10]); gout development [11]; brain abnormalities [12]; stunting in preschool children [13] and low bone mineral density [14].

Our previous data suggested that low ficolin-2 concentration (determined in cord serum) is associated with prematurity, low birthweight and perinatal infections [15]. Recently, we reported for the first time a relationship between SNPs of the *FCN2* gene 3′-untranslated region (3′UTR) and very low (≤1500 g) birthweight as well as early onset of infection and pneumonia in preterm newborns [4]. Furthermore, two of the 3′UTR polymorphisms (rs4521835 and rs73664188) influenced ficolin-2 concentration in cord sera [4]. Here, we report associations of the aforementioned promoter region polymorphism with short gestational age and very low birthweight in the same cohort.

## 2. Results

The frequencies of genotypes corresponding to SNPs at positions −986 (rs3124952), −602 (rs3124953), −64 (rs7865453) and −4 (rs17514136) of the *FCN2* gene as well as minor allele frequencies (MAF) in preterm neonates are listed in Table 1. Each SNP adhered to Hardy–Weinberg expectations (*p* > 0.01, details are given in Supplementary Table S1). As mentioned, they created two haplotype blocks (rs3124952 and rs3124953; rs7865453 and rs17514136) (Supplementary Figure S1). None appeared associated with incidence of RDS, early- or late-onset perinatal infections, sepsis or pneumonia (Supplementary Table S2). However, major (A) alleles at positions −986 and −64 as well as A/A homozygosity for both polymorphisms were significantly less frequent among babies born with very low birthweight (VLBW) (≤1500 g) when compared with the corresponding reference group. Those relationships remained significant after multiple logistic regression analysis (Table 2). Moreover, lower frequency of G/G (minor allele) homozygosity at position −4 was found to be associated not only with low body mass but also with shorter gestation (Table 2). Those associations were however not confirmed by multiple logistic regression (*p* > 0.05). It should be stressed that minor variants at both −986 and −64 positions are associated with lower ficolin-2 concentration in serum compared to A alleles while the G variant at −4 is associated with a higher *FCN2* gene expression level. VLBW was not significantly related to the sex of newborns, although a trend towards its higher incidence in girls was observed (13.5% vs. 8.7%, *p* = 0.087). The numbers of females and males born at gestational age <33 weeks did not differ significantly (23.3% vs. 18.9%, *p* = 0.22).

Eleven promoter haplotypes were identified with the help of Haploview software, including three with MAF > 0.1 and two with a marginally lower value (Table 3). Their frequencies, depending on gestational age and body mass at birth, are shown in Table 4. The most common haplotype (AGAG) may be considered protective from very low birthweight, whilst the fifth most frequent (GGCA) seemed to have the opposite effect, confirmed by multiple logistic regression analysis as well (Table 4). That effect appeared independent of gestational age: the frequency of the GGCA variant was significantly higher among babies with very low birthweight compared with the corresponding reference group, born at <33 [0.146 vs. 0.048, OR = 3.43, 95% CI (1.23–9.54), *p* = 0.021] as well as ≥33 weeks [0.214 vs. 0.096, OR = 2.57, 95% CI (1.01–6.53), *p* = 0.04], respectively. Again, no association of any haplotype with RDS, infections, sepsis, pneumonia (Supplementary Table S3) or shorter gestational age was noted (Table 4).

Further analysis using PHASE software revealed thirty diplotypes, although the frequency of half of them was less than 1% (Table 5). Interestingly, the most common one, AGAG/GGAA, was associated with adverse events (shorter gestational age and very low birthweight) (Table 6). However, after multiple logistic regression analysis, that relationship lost statistical significance (*p* > 0.05). Furthermore, diplotypes possessing the GGCA haplotype (5, 6, 11, 14, 20, 24, 30, see Table 5) were significantly more frequent among neonates with birthweight ≤1500 g [17/55 (30.9%)] compared with the corresponding reference group [76/443 (17.2%)] [OR = 2.16, *p* = 0.0175, 95% CI (1.16–4.03)]. In contrast, AGAG homozygosity was found to be protective from lower body mass (Table 6), although that association lost significance in multiple logistic regression analysis (*p* > 0.05). There was also a trend for more babies with AGAG homozygosity to be born after 33 weeks of gestation (Table 6). None of the ten most common diplotypes was associated with RDS, infections, sepsis or pneumonia (Supplemetary Table S4).

We investigated the relationship of the ten most common diplotypes with ficolin-2 concentration in cord serum. The Kruskal–Wallis ANOVA revealed significant differences among genotypes (Supplementary Figure S2). Diplotype 2 (AGAG/AAAA, 2748 ng/mL) had a higher median than the others, although the difference between it and diplotype 3 did not quite reach statistical significance (*p* = 0.067). Diplotype 5 (AGAG/GGCA) had the lowest median at 1415 ng/mL (Supplementary Figure S2). However, we found no clinical associations with either diplotype 2 or 5 (at least when analysed individually) (Table 6). It is worth noting that, in general, diplotypes including the GGCA haplotype were associated with relatively low serum ficolin-2. Nevertheless, wide ranges were found for most diplotypes (Table 5; Supplementary Figure S2).

## 3. Discussion

Genome-wide association studies (GWAS) have enabled identification of a variety of loci/SNPs associated with gestational age and birthweight. The majority of reports concerns maternal genome analysis while data from newborns are relatively scarce. Tiensuu et al. [16] found an association of rs116461311 polymorphism (*SLIT2* gene, encoding slit guidance ligand 2) with spontaneous preterm birth. Furthermore, they observed higher expression of the SLIT2 protein and its receptor ROBO1 in placentas from preterm deliveries compared with those from term births. The SLIT2-ROBO1 signaling pathway is involved, among others, in regulation of expression of genes associated with inflammation [16]. Rappoport et al. [17], based on the analysis of >2 million SNPs in five populations, reported only two loci to be significantly related to prematurity: rs17591250 and rs1979081 in African and American populations, respectively. Later, Huusko et al. [18], based on GWAS and other methods, identified genes encoding heat shock proteins and nuclear receptors (*SEC63*, *HSPA1L*, *SACS*, *RORA*, and *AR*) to be associated with spontaneous preterm birth. Using another approach, whole exome sequencing (WES), Modi et al. [19] proposed candidate genes in which mutations were found to be risk factors for preterm premature rupture of membranes (pPROM), one of the major causes of prematurity. Those genes (*CARD6*, *CARD8*, *DEFB1*, *FUT2*, *MBL2*, *NLP10*, *NLRP12* and *NOD2*) are involved in host defence. Interestingly, data concerning association of the *MBL2* gene [encoding mannose-binding lectin (MBL), structurally and functionally related to ficolins] polymorphisms with preterm birth are contradictory. Several reports suggested MBL deficiency to be a risk factor [20,21,22]. In contrast, Swierzko et al. [15] found high MBL concentration/activity-conferring genotypes to be associated with prematurity.

A GWAS analysis concerning birthweight performed by Luo et al. [23] identified a variety of loci in both maternal and fetal genomes potentially affecting this parameter in four populations (Afro-Caribbean, European, Hispanic and Thai). Extensive meta-analyses [24,25], identified a variety of loci where fetal genotype was associated with birthweight and found their associations with height, body-mass index and some metabolic diseases in adulthood.

The role of ficolin-2 in neonatal health and disease has not been studied extensively. Kilpatrick et al. [26] first reported lower concentrations in cord sera compared with sera from adult donors. Furthermore, ficolin-2 levels correlated positively with both gestational age and birthweight. Later, Swierzko et al. [15] confirmed those findings with a large (>1800) cohort of newborns. An association of low ficolin-2 with prematurity was further reported by Schlapbach et al. [27], Sallenbach et al. [28] and Kilpatrick et al. [3]. However, Briana et al. [29] observed no impact of ficolin-2 concentration on intrauterine growth restriction in full-term newborns. 

Ficolin-2 is known to recognise a variety of pathogens, including group B streptococci, pneumococci and enteroaggregative *E. coli*, that can cause severe infections in newborns and/or infants [30,31,32,33]. Cord serum concentrations of this protein <1 µg/mL were found significantly more often among preterm babies with perinatal infections, compared with gestational-age-matched controls [15]. Later, we reported markedly lower ficolin-2 levels in neonates suffering from perinatal sepsis versus those without infections before hospital discharge [34]. On the other hand, Schlapbach et al. [27] did not find such an association. 

Much less data concerning the role of *FCN2* gene polymorphisms, including those affecting ficolin-2 concentration, in neonates has been published to date. Our previous report [3] demonstrated that the genotype A/G-G/G-A/A-A/A-A/G-C/T-G/G (corresponding to SNPs at positions −986, −602, −557, −64, −4, +6369 and +6424) was the most common among Polish newborns. That genotype corresponds to the commonest AGAG/GGAA diplotype described in this paper on the basis of analysis with PHASE software (version 2.2.1.) (Table 5). Our current data from a large cohort of preterm babies found it to be associated with a relatively high risk of short (<33 weeks) gestational age and very low (≤1500 g) body mass at birth. 

It should be stressed that AGAG/AGAG homozygosity seems protective from very low birthweight (Table 6). It differs from the AGAG/GGAA in one haplotype only, by possessing a major allele (A) at rs3124952 (−986) and a minor one (G) at rs17514136 (−4), both related to higher ficolin-2 concentration. The possible causal relationship between ficolin-2 concentration and outcome is strengthened by the association of the GGCA haplotype (generally associated with low serum ficolin-2) with very low birthweight (Table 4).

Furthermore, when each polymorphic site was analysed separately, homozygosity for major alleles at −986 (rs3124952) and −64 (rs7865453) and minor allele at −4 (rs17514136), associated with higher ficolin-2 levels, appears protective from very short gestational age or very low birthweight (Table 2). Although no impact of the SNPs, haplo- or diplotypes investigated here on such adverse effects of prematurity as perinatal infections, sepsis, pneumonia or RDS was found (Supplementary Tables S2–S4), it does not exclude a possible influence of low ficolin-2 on such complications. The *FCN2* gene is highly polymorphic and the concentration of its product depends on the interplay between SNPs localised to the promoter, exon 8 and 3′UTR regions and, possibly, epigenetic mechanisms. Furthermore, the ficolin-2 protein has several active sites and genetic changes can influence both concentration and activity, making for a very complex situation. Individuals may possess genetic variants that influence the ficolin-2 level in opposite directions [35]. Together with results published previously by ourselves and others, the data presented here are consistent with the view that ficolin-2 is an important factor for fetal development and neonatal immunity. The most important message from the data presented here is that an association of the *FCN2* gene promoter polymorphisms with very low birthweight may have potentially severe clinical consequences not only in the neonatal period but also during later life. Supplementary Figure S3 shows an interplay between four investigated SNPs, corresponding haplo- and diplotypes, ficolin-2 concentrations and the aforementioned adverse effects of prematurity.

## 4. Materials and Methods

### 4.1. Cohort

The study group comprised 501 Polish preterm newborns born in the Department of Newborns’ Infectious Diseases (University of Medical Sciences, Poznań, Poland), Department of Neonatology (Medical University of Gdańsk, Gdańsk, Poland) and Department of Perinatology (Medical University of Łódź, Łódź, Poland) [4]. Among them, 105 were born at gestational age < 33 weeks (mean: 30.3 ± 1.9; range: 24–32) and 396 were born between the 33rd and 37th week of gestation (mean: 35 ± 1.1). Fifty-five had very low birthweight (≤1500 g, according to WHO International Classification of Diseases). A total of 323 newborns came from singleton pregnancies, 172 from 97 twin pregnancies (in 22, material from only one sibling was collected) and 6 from 2 triple pregnancies. Data concerning the *FCN2* gene 3′UTR polymorphisms, concentrations of ficolin-2 in cord sera and their clinical associations were published recently [4]. However, 3 subjects were excluded from current analyses due to incomplete results of promoter SNP analysis. As mentioned, promoter SNPs analysed here were previously reported to form 2 haplotype blocks: one created by rs3124952 and rs3124953, and another, by rs7865453 and rs17514136 [4]. The study was approved by the corresponding local ethics committees: Bioethics Committee of The Karol Marcinkowski Poznań University of Medical Sciences, Independent Bioethics Committee for Scientific Research at The Medical University of Gdańsk, Bioethics Committee of The Medical University of Łódź. Written informed parental consent was obtained. This work conforms to the provisions of the Declaration of Helsinki.

### 4.2. Blood Samples and DNA Isolation

Cord blood samples for genomic DNA isolation were taken consecutively into tubes with sodium citrate and stored at −80 °C. DNA was isolated using GeneMATRIX Quick Blood Purification Kit (EURx Ltd. Gdańsk, Poland), according to the manufacturer’s protocol. Blood for serum isolation was placed in tubes containing clot activator. Samples were kept at −80 °C.

### 4.3. Determination of the FCN2 Gene Polymorphisms

Promoter polymorphisms at positions −986 (rs3124952, A > G) and −602 (rs3124953, G > A) were investigated by PCR-RFLP analysis, according to the procedures published by Metzger et al. [36]. SNPs at positions −64 (rs7865453, A > C) and −4 (rs17514136, A > G) were determined using allele-specific PCR or PCR-RFLP, respectively, as described by Szala et al. [37], with minor modifications.

### 4.4. Determination of Ficolin-2 Concentration in Cord Sera

Ficolin-2 concentrations in cord serum samples were determined in TRIFMA as described by Świerzko et al. [38], using specific mAb (ABS 005-16, BioPorto Diagnostics, Denmark) for coating and another biotinylated mAb (GN4, Hycult Biotech, Uden, The Netherlands) and Eu^3+^-labelled streptavidin (Perkin Elmer, Waltham, MA, USA) for detection.

### 4.5. Statistical Analysis

Linkage disequilibrium (LD) and haplotype block analysis were performed by Haploview 4.2 software (http://www.broad.mit.edu/mpg/haploview/, accessed on 30 June 2022). LD analysis was performed for each pair of polymorphisms using D’ and r^2^, indicating the amount of LD between two genetic loci. Haplotype block identification was performed based on the Four Gamete Rule. The PHASE software (http://stephenslab.uchicago.edu/phase/download.html, accessed on 30 June 2022; version 2.1.1.) was used for diplotype reconstruction from genotype data. The frequencies of genotypes were compared by Fischer’s exact (two-tailed) test. Ficolin-2 concentrations were compared with Kruskal–Wallis ANOVA and Mann–Whitney *U* tests. The Statistica (version 13.3, TIBCO Software) and SigmaPlot (version 12, Systat Software) software packages were used for data management and statistical calculations. Odds ratio was calculated using online MedCalc software (https://www.medcalc.org, accessed on 30 June 2022). *p* values < 0.05 were considered statistically significant. 

## Figures and Tables

**Table 1:** Distribution of genotypes associated with *FCN2* gene promoter polymorphisms in preterm newborns (n = 501).

Polymorphism	Genotype	N	%	MAF
rs3124952 −986 A > G	A/A	165	32.9	0.386
	A/G	265	52.9	
	G/G	61	12.2	
rs3124953 −602 G > A	G/G	314	62.7	0.204
	G/A	170	33.9	
	A/A	17	3.4	
rs7865453 −64 A > C	A/A	390	77.8	0.115
	A/C	107	21.4	
	C/C	4	0.8	
rs17514136 −4 A > G	A/A	204	40.7	0.347
	A/G	243	48.5	
	G/G	51	10.2	

**Table 2:** Distribution of genotypes associated with *FCN2* gene promoter polymorphisms in preterm newborns, depending on gestational age and birthweight.

Polymorphism	Genotype	Gestational Age (Weeks)		Birthweight (g)	
		<33	≥33	≤1500	>1500
		N (%)	N (%)	N (%)	N (%)
rs3124952 −986 A > G	A/A	32 (30.5)	143 (36.1)	7 (12.7) ^2^	168 (37.9)
	A/G	61 (58.1)	204 (51.5)	39 (70.9)	223 (50.3)
	G/G	12 (11.4)	49 (12.4)	9 (16.4)	52 (11.7)
rs3124953 −602 G > A	G/G	69 (65.7)	245 (61.9)	35 (63.6)	276 (62.3)
	G/A	32 (30.5)	138 (34.8)	20 (36.4)	150 (33.9)
	A/A	4 (5.8)	13 (3.3)	0 (0)	17 (3.8)
rs7865453 −64 A > C	A/A	83 (79)	307 (77.5)	36 (65.5) ^3^	352 (79.5)
	A/C	22 (21)	85 (21.5)	18 (32.7)	88 (19.9)
	C/C	0 (0)	4 (1)	1 (1.8)	3 (0.7)
rs17514136 −4 A > G	A/A	38 (36.2)	166 (41.9)	26 (47.3)	177 (40)
	A/G	62 (59)	184 (46.5)	29 (52.7)	215 (48.5)
	G/G	5 (4.8) ^1^	46 (11.6)	0 (0) ^4^	51 (11.5)

^1^—OR = 0.38, 95% CI (0.15—0.98), *p* = 0.0447. ^2^—OR = 0.24. 95% CI (0.11—0.54), *p* = 0.0029; OR = 0.25, 95% CI (0.11–0.61), *p* = 0.002, after multiple logistic regression; A allele frequency: 0.482 vs. 0.631; OR = 0.54. 95% CI (0.37—0.81), *p* = 0.0035. ^3^—OR = 0.49. 95% CI (0.27—0.89), *p* = 0.0244; OR = 0.33, 95% CI (0.15–0.71), *p* = 0.005, after multiple logistic regression; A allele frequency: 0.818 vs. 0.894; OR = 0.53, 95% CI (0.31—0.91), *p* = 0.0254. ^4^—OR = 0.07, 95% CI (0.004—1.13), *p* = 0.0034.

**Table 3:** Frequencies of haplotypes identified in preterm newborns (n = 501).

Haplotype	N	Frequency
AGAG	307	0.306
GGAA	244	0.244
AAAA	192	0.192
AGAA	99	0.099
GGCA	98	0.098
GGAG	41	0.041
AGCA	6	0.006
AACA	5	0.005
GACA	4	0.004
GAAA	4	0.004
AGCG	2	0.002

**Table 4:** Frequencies of the most common haplotypes in preterm newborns, depending on gestational age and birthweight.

Haplotype	Gestational Age (Weeks)				Birthweight (g)			
	<33		≥33		≤1500		>1500	
	N	Frequency ^1^	N	Frequency ^1^	N	Frequency ^1^	N	Frequency ^1^
AGAG	65	0.31	242	0.306	24 ^2^	0.218	281	0.317
GGAA	57	0.271	187	0.236	35	0.318	207	0.234
AAAA	38	0.181	154	0.194	18	0.164	174	0.196
AGAA	20	0.095	79	0.1	7	0.064	91	0.103
GGCA	19	0.09	79	0.1	18 ^3^	0.164	79	0.089

^1^—haplotype frequency among newborns born at GA <33/≥33 weeks; with birthweight ≤1500/>1500 g, respectively. ^2^—OR = 0.6, 95% CI (0.37—0.97), *p* = 0.0369; OR = 0.5, 95% CI (0.25–0.99), *p* = 0.047, after multiple logistic regression. ^3^—OR = 2.95, 95% CI (1.15—3.48), *p* = 0.0249; OR = 3.53, 95% CI (1.57–7.97), *p* = 0.002, after multiple logistic regression.

**Table 5:** Frequencies of diplotypes identified in preterm newborns (n = 501).

Diplotype		N	%	Ficolin-2 Concentration (ng/mL)	
				Median	Range (n)
1	AGAG/GGAA	94	18.8	1761	237–5068 (80)
2	AGAG/AAAA	60	12	2748	481–5235 (56)
3	AAAA/GGAA	46	9.2	2327	803–5166 (39)
4	AGAG/AGAG	41	8.2	1950	632–5299 (38)
5	AGAG/GGCA	33	6.6	1415	430–4081 (30)
6	AAAA/GGCA	29	5.8	1900	504–5644 (27)
7	GGAA/GGAA	28	5.6	1743	153–4772 (27)
8	AGAA/GGAA	27	5.4	2192	372–5408 (25)
9	AGAA/AGAG	24	4.8	1785	479–4426 (22)
10	AAAA/GGAG	21	4.2	2159	407–4199 (19)
11	GGAA/GGCA	17	3.4	1098	242–2157 (16)
12	AGAA/AGAA	16	3.2	2165	853–5481 (16)
13	AAAA/AAAA	15	3	2323	690–4038 (15)
14	AGAA/GGCA	8	1.6	1479	480–3063 (8)
15	GGAG/GGAG	6	1.2	2134	706–4165 (5)
16	AGAA/AAAA	4	0.8	3240	1455–4954 (4)
17	AGAA/AGCA	4	0.8	2562	1737–2733 (3)
18	AGAG/AACA	4	0.8	2495	1698–2919 (4)
19	AGAG/GACA	4	0.8	1105	652–1563 (4)
20	GGCA/GGCA	4	0.8	681	331–947 (3)
21	AGAG/GGAG	3	0.6	2120	1387–4756 (3)
22	GGAA/GGAG	3	0.6	1745	312–2195 (3)
23	AGAG/AGCG	2	0.4	2002	520–3483 (2)
24	GGAG/GGCA	2	0.4	907	387–1426 (2)
25	AAAA/AACA	1	0.2	2210	2210 (1)
26	AGAG/AGCA	1	0.2	2221	2221 (1)
27	AGCA/AAAA	1	0.2	239	239 (1)
28	GAAA/GAAA	1	0.2	3531	3531 (1)
29	GGAA/GAAA	1	0.2	2194	2194 (1)
30	GGCA/GAAA	1	0.2	937	937 (1)

**Table 6:** Frequencies of the most common diplotypes in preterm newborns, depending on gestational age and birthweight.

Diplotype		Gestational Age (Weeks)				Birthweight (g)			
		<33		≥33		≤1500		>1500	
		N	% ^1^	N	% ^1^	N	% ^1^	N	% ^1^
1	AGAG/GGAA	28 ^2^	26.7	66	16.7	16 ^3^	29.1	77	17.4
2	AGAG/AAAA	11	10.5	49	12.4	3	5.5	57	12.9
3	AAAA/GGAA	9	8.6	37	9.3	7	12.7	39	8.8
4	AGAG/AGAG	5	4.8	36	9.1	0	0 ^4^	41	9.3
5	AGAG/GGCA	6	5.7	27	6.8	3	5.5	30	6.8
6	AAAA/GGCA	5	4.8	24	6.1	5	9.1	24	5.4
7	AGAA/GGAA	7	6.7	20	5.1	2	3.6	25	5.6
8	GGAA/GGAA	4	3.8	24	6.1	3	5.5	25	5.6
9	AGAA/AGAG	9	8.6	15	3.8	1	1.8	22	5
10	AAAA/GGAG	4	3.8	17	4.3	2	3.6	19	4.3

^1^—percentages of diplotype 1–10 carriers among newborns born at GA <33/≥33 weeks; with birthweight ≤1500/>1500 g, respectively. ^2^—OR = 1.82, 95% CI (1.1—3.02), *p* = 0.0243. ^3^—OR = 1.95, 95% CI (1.04—3.67), *p* = 0.0434. ^4^—OR = 0.087, 95% CI (0.005—1.44), *p* = 0.0155.

## Data Availability

The data are available from the corresponding author on reasonable request.

## Supplementary Materials

The following supporting information can be downloaded at https://www.mdpi.com/article/10.3390/ijms232315336/s1. Table S1. Hardy–Weinberg expectation statistics for investigated *FCN2* gene promoter polymorphisms. SNPs were considered to adhere to Hardy–Weinberg expectations when *p* > 0.01. Table S2. Distribution of genotypes associated with *FCN2* gene promoter polymorphisms in preterm newborns, depending on incidence of respiratory distress syndrome, early-onset infection, pneumonia and sepsis. None of the associations analysed was significant (*p* > 0.05). Table S3. Frequencies of the most common haplotypes in preterm newborns, depending on incidence of respiratory distress syndrome, early-onset infection, pneumonia and sepsis. None of the associations analysed was significant (*p* > 0.05). Table S4. Frequencies of the most common diplotypes in preterm newborns, depending on incidence of respiratory distress syndrome, early-onset infection, pneumonia and sepsis. None of the associations analysed was significant (*p* > 0.05). Supplementary Figure S1: Linkage disequilibrium analysis of promoter rs3124952 (−986 A > G), rs3124953 (−602 G > A), rs7865453 (−64 A > C) and rs17514136 (−4 A > G) *FCN2* single nucleotide polymorphisms. The numbers in the grid refer to D’ (**A**) and r^2^ (**B**) parameters of the given pairs of SNPs. Bolded triangles show haplotype blocks identified using the four gamete rule test. Supplementary Figure S2: Individual concentrations of ficolin-2 in cord sera from preterm newborns, corresponding to the ten most common *FCN2* gene promoter diplotypes. Blue bars represent median values (given below the graph in bold). Medians related to diplotypes 2 (the highest) and 5 (the lowest one) were compared with the remaining values using a Mann–Whitney U test. Corresponding *p*-values are given below the graph in red and blue, respectively. Diplotypes: 1—AGAG/GGAA; 2—AGAG/AAAA; 3—AAAA/GGAA; 4—AGAG/AGAG; 5—AGAG/GGCA; 6—AAAA/GGCA; 7—GGAA/GGAA; 8—AGAA/GGAA; 9—AGAA/AGAG; 10—AAAA/GGAG. Supplementary Figure S3: **A:** Scheme of the *FCN2* gene with investigated promoter polymorphic sites. Alleles associated with higher gene expression are marked in green and those with lower, in red. Exons 1–8 are shown as blue rectangles. **B:** Genotypes corresponding to polymorphic sites, most common haplotypes, diplotypes and median ficolin-2 concentrations in cord sera (ng/mL), related to demonstrated promoter diplotypes. Alleles corresponding to particular sites associated with higher gene expression are marked in green and those with lower, in red. Median ficolin-2 levels higher than the median for the whole cohort are marked in green and those lower, in red. Genotypes: A/A (−986), A/A (−64), G/G (−4), all corresponding to relatively high FCN2 gene expression; the related AGAG haplotype and AGAG/AGAG diplotype were associated with lower risk of very low birthweight (green boxes). Furthermore, the G/G variant at −4 corresponds to a lower risk of birth at gestational age <33 weeks (blue box). The GGCA haplotype (all alleles related to lower gene expression) was associated with a higher risk of VLBW (red box) while the AGAG/GGAA diplotype was associated with a higher risk of both VLBW and GA < 33 weeks (red and orange boxes).

## References

[1] Hummelshoj T., Munthe-Fog L., Madsen H.O., Fujita T., Matsushita M., Garred P. (2005). Polymorphisms in the FCN2 gene determine serum variation and function of Ficolin-2. Hum. Mol. Genet..

[2] Cedzynski M., Nuytinck L., Atkinson A.P.M., Swierzko A.S., Zeman K., Szemraj J., Szala A., Turner M.L., Kilpatrick D.C. (2007). Extremes of l-ficolin concentration in children with recurrent infections are associated with single nucleotide polymorphisms in the *FCN2* gene. Clin. Exp. Immunol..

[3] Kilpatrick D.C., Swierzko A.S., Matsushita M., Domzalska-Popadiuk I., Borkowska-Klos M., Szczapa J., Cedzynski M. (2013). The relationship between FCN2 genotypes and serum ficolin-2 (L-ficolin) protein concentrations from a large cohort of neonates. Hum. Immunol..

[4] Świerzko A.S., Jarych D., Gajek G., Chojnacka K., Kobiela P., Kufelnicka-Babout M., Michalski M., Sobczuk K., Szala-Poździej A., Matsushita M. (2021). Polymorphisms of the FCN2 Gene 3′UTR Region and Their Clinical Associations in Preterm Newborns. Front. Immunol..

[5] Thiel S., Gadjeva M. (2009). Humoral Pattern Recognition Molecules: Mannan-Binding Lectin and Ficolins. Adv. Exp. Med. Biol..

[6] Endo Y., Matsushita M., Fujita T. (2015). New Insights into the Role of Ficolins in the Lectin Pathway of Innate Immunity. Int. Rev. Cell. Mol. Biol..

[7] Świerzko A.S., Cedzyński M. (2020). The Influence of the Lectin Pathway of Complement Activation on Infections of the Respiratory System. Front. Immunol..

[8] Statistics Poland (2022). Demographic Yearbook of Poland, 2021.

[9] Heron M. (2021). Deaths: Leading causes for 2019. Natl. Vital Stat. Rep..

[10] Van de Pol C., Allegaert K. (2020). Growth patterns and body composition in former extremely low birth weight (ELBW) neonates until adulthood: A systematic review. Eur. J. Pediatr..

[11] Dehlin M., Jacobsson L.T.H. (2022). Association between perinatal factors and future risk for gout—A nested case-control study. Arthritis Res. Ther..

[12] Kuula J., Martola J., Hakkarainen A., Räikkönen K., Savolainen S., Salli E., Hovi P., Björkqvist J., Kajantie E., Lundbom N. (2022). Brain Volumes and Abnormalities in Adults Born Preterm at Very Low Birth Weight. J. Pediatr..

[13] Halli S.S., Biradar R.A., Prasad J.B. (2022). Low Birth Weight, the Differentiating Risk Factor for Stunting among Preschool Children in India. Int. J. Environ. Res. Public Health.

[14] Sandboge S., Kuula J., Björkqvist J., Hovi P., Mäkitie O., Kajantie E. (2022). Bone mineral density in very low birthweight adults—A sibling study. Paediatr. Peérinat. Epidemiol..

[15] Swierzko A.S., Atkinson A.P., Cedzynski M., MacDonald S.L., Szala A., Domzalska-Popadiuk I., Borkowska-Klos M., Jopek A., Szczapa J., Matsushita M. (2009). Two factors of the lectin pathway of complement, l-ficolin and mannan-binding lectin, and their associations with prematurity, low birthweight and infections in a large cohort of Polish neonates. Mol. Immunol..

[16] Tiensuu H., Haapalainen A.M., Karjalainen M.K., Pasanen A., Huusko J.M., Marttila R., Ojaniemi M., Muglia L.J., Hallman M., Ramet M. (2019). Risk of spontaneous preterm birth and fetal growth associates with fetal SLIT2. PLoS Genet..

[17] Rappoport N., Toung J., Hadley D., Wong R.J., Fujioka K., Reuter J., Abbott C.W., Oh S., Hu D., Eng C. (2018). A genome-wide association study identifies only two ancestry specific variants associated with spontaneous preterm birth. Sci. Rep..

[18] Huusko J.M., Karjalainen M.K., Graham B.E., Zhang G., Farrow E.G., Miller N.A., Jacobsson B., Eidem H.R., Murray J.C., Bedell B. (2018). Whole exome sequencing reveals HSPA1L as a genetic risk factor for spontaneous preterm birth. PLoS Genet..

[19] Modi B.P., Teves M.E., Pearson L.N., Parikh H.I., Haymond-Thornburg H., Tucker J.L., Chaemsaithong P., Gomez-Lopez N., York T.P., Romero R. (2017). Mutations in fetal genes involved in innate immunity and host defense against microbes increase risk of preterm premature rupture of membranes (PPROM). Mol. Genet. Genom. Med..

[20] Frakking F.N.J., Brouwer N., Zweers D., Merkus M.P., Kuijpers T.W., Offringa M., Dolman K.M. (2006). High prevalence of mannose-binding lectin (MBL) deficiency in premature neonates. Clin. Exp. Immunol..

[21] Bodamer O.A., Mitterer G., Maurer W., Pollak A., Mueller M.W., Schmidt W.M. (2006). Evidence for an association between mannose-binding lectin 2 (MBL2) gene polymorphisms and pre-term birth. Genet. Med..

[22] da Silva L.V.C., Javorski N., Brandão L.A.C., Lima M.D.C., Crovella S., Eickmann S.H. (2018). Influence of *MBL2* and *NOS3* polymorphisms on spontaneous preterm birth in North East Brazil: Genetics and preterm birth. J. Matern. Neonatal Med..

[23] Liu X., Cui Y. (2016). A Genome-wide Association Analysis in Four Populations Reveals Strong Genetic Heterogeneity for Birth Weight. Curr. Genom..

[24] Horikoshi M., Yaghootkar H., Mook-Kanamori D.O., Sovio U., Taal H.R., Hennig B.J., Bradfield J.P., Pourcain B.S., Evans D.M., Charoen P. (2012). New loci associated with birth weight identify genetic links between intrauterine growth and adult height and metabolism. Nat. Genet..

[25] Thompson W.D., Beaumont R.N., Kuang A., Warrington N.M., Ji Y., Tyrrell J., Wood A.R., Scholtens D.M., Knight B.A., Evans D.M. (2021). Fetal alleles predisposing to metabolically favorable adiposity are associated with higher birth weight. Hum. Mol. Genet..

[26] Kilpatrick D.C., Fujita T., Matsushita M. (1999). P35, an opsonic lectin of the ficolin family, in human blood from neonates, normal adults, and recurrent miscarriage patients. Immunol. Lett..

[27] Schlapbach L.J., Mattmann M., Thiel S., Boillat C., Otth M., Nelle M., Wagner B., Jensenius J.C., Aebi C., Christoph A. (2010). Differential Role of the Lectin Pathway of Complement Activation in Susceptibility to Neonatal Sepsis. Clin. Infect. Dis..

[28] Sallenbach S., Thiel S., Aebi C., Otth M., Bigler S., Jensenius J.C., Schlapbach L., Ammann R.A. (2011). Serum concentrations of lectin-pathway components in healthy neonates, children and adults: Mannan-binding lectin (MBL), M-, L-, and H-ficolin, and MBL-associated serine protease-2 (MASP-2). Pediatr. Allergy Immunol..

[29] Briana D.D., Liosi S., Gourgiotis D., Boutsikou M., Baka S., Marmarinos A., Hassiakos D., Malamitsi-Puchner A. (2012). The potential role of the lectin pathway of complement in the host defence of full-term intrauterine growth restricted neonates at birth. J. Matern. Neonatal Med..

[30] Aoyagi Y., Adderson E.E., Rubens C.E., Bohnsack J.F., Min J.G., Matsushita M., Fujita T., Okuwaki Y., Takahashi S. (2008). L-Ficolin/Mannose-Binding Lectin-Associated Serine Protease Complexes Bind to Group B Streptococci Primarily through *N*-Acetylneuraminic Acid of Capsular Polysaccharide and Activate the Complement Pathway. Infect. Immun..

[31] Fujieda M., Aoyagi Y., Matsubara K., Takeuchi Y., Fujimaki W., Matsushita M., Bohnsack J.F., Takahashi S. (2012). L-Ficolin and Capsular Polysaccharide-Specific IgG in Cord Serum Contribute Synergistically to Opsonophagocytic Killing of Serotype III and V Group B Streptococci. Infect. Immun..

[32] Brady A.M., Calix J.J., Yu J., Geno K.A., Cutter G.R., Nahm M.H. (2014). Low Invasiveness of Pneumococcal Serotype 11A Is Linked to Ficolin-2 Recognition of O-acetylated Capsule Epitopes and Lectin Complement Pathway Activation. J. Infect. Dis..

[33] Sørensen C.A., Rosbjerg A., Jensen B.H., Krogfelt K.A., Garred P. (2018). The Lectin Complement Pathway Is Involved in Protection Against Enteroaggregative Escherichia coli Infection. Front. Immunol..

[34] Świerzko A.S., Szala-Poździej A., Kilpatrick D.C., Sobociński M., Chojnacka K., Sokołowska A., Michalski M., Mazerant K., Jensenius J.C., Matsushita M. (2016). Components of the lectin pathway of complement activation in paediatric patients of intensive care units. Immunobiology.

[35] Kilpatrick D.C., Świerzko A.S., Sobociński M., Krajewski W., Chojnacka K., Szczapa J., Cedzyński M. (2015). Can ficolin-2 (L-ficolin) insufficiency be established by a single serum protein measurement?. Int. J. Immunogenet..

[36] Metzger M.-L., Michelfelder I., Goldacker S., Melkaoui K., Litzman J., Guzman D., Grimbacher B., Salzer U. (2015). Low ficolin-2 levels in common variable immunodeficiency patients with bronchiectasis. Clin. Exp. Immunol..

[37] Szala A., Swierzko A.S., Cedzynski M. (2013). Cost-effective procedures for genotyping of human FCN2 gene single nucleotide polymorphisms. Immunogenetics.

[38] Swierzko A.S., Michalski M., Sokołowska A., Nowicki M., Szala-Poździej A., Eppa Ł., Mitrus I., Szmigielska-Kapłon A., Sobczyk-Kruszelnicka M., Michalak K. (2020). Associations of Ficolins With Hematological Malignancies in Patients Receiving High-Dose Chemotherapy and Autologous Hematopoietic Stem Cell Transplantations. Front. Immunol..

