# Supplementary material for: Association of the FCN2 Gene Promoter Region Polymorphisms with Very Low Birthweight in Preterm Neonates
Source: Int J Mol Sci. 2022 Dec 5;23(23):15336. doi: 10.3390/ijms232315336 (PMC9740280; doi:10.3390/ijms232315336)
Supplement: Supplementary file 1 [file ijms-23-15336-s001.zip › Supplementary Material.pdf]

Supplementary Table S1. Hardy-Weinberg expectation statistics for investigated *FCN2* gene promoter polymorphisms. SNP was considered to adhere to Hardy-Weinberg expectation when p>0.01.

Polymorphism	p value
rs3124952 (-986 A>G)	0.0444
rs3124953 (-602 G>A)	0.2742
rs7865453 (-64 A>C)	0.4234
rs17514136 (-4 A>G)	0.0807

Supplementary Table S2. Distribution of genotypes associated with *FCN2* gene promoter polymorphisms in preterm newborns, depending on incidence of respiratory distress syndrome, early-onset infection, pneumonia and sepsis. None of associations analysed apperaed significant (p>0.05).

Polymorphism	Genotype	Respiratory distress syndrome		Early-onset infection		Pneumonia		Sepsis	
		Yes	No	Yes	No	Yes	No	Yes	No
		N (%)	N (%)	N (%)	N (%)	N (%)	N (%)	N (%)	N (%)
rs3124952 -986 A>G	A/A	41 (31.8)	135 (36.6)	35 (33)	141 (35.8)	18 (38.3)	158 (35)	4 (25)	172 (35.5)
	A/G	71 (55)	190 (51.5)	59 (55.7)	204 (51.8)	27 (57.4)	235 (52)	11 (68.8)	252 (52.1)
	G/G	17 (13.2)	44 (11.9)	12 (11.3)	49 (12.4)	2 (4.3)	59 (13)	1 (6.3)	60 (12.4)
rs3124953 -602 G>A	G/G	82 (63.6)	229 (62.1)	63 (59.4)	250 (63.5)	24 (51.1)	288 (63.7)	8 (50)	305 (63)
	G/A	44 (34.1)	126 (34.1)	38 (35.8)	132 (33.5)	20 (42.6)	150 (33.2)	8 (50)	162 (33.5)
	A/A	3 (2.3)	14 (3.8)	5 (4.7)	12 (3)	3 (6.4)	14 (3.1)	0 (0)	17 (3.5)
rs7865453 -64 A>C	A/A	96 (74.4)	292 (79.1)	81 (76.4)	309 (78.4)	40 (85.1)	349 (77.2)	15 (93.8)	375 (77.5)
	A/C	33 (25.6)	73 (19.8)	24 (22.6)	82 (20.8)	7 (14.9)	99 (21.9)	1 (6.3)	105 (21.7)
	C/C	0 (0)	4 (1.1)	1 (0.9)	3 (0.8)	0 (0)	4 (0.9)	0 (0)	4 (0.8)
rs17514136 -4 A>G	A/A	48 (37.2)	155 (42)	44 (41.5)	159 (40.4)	20 (42.6)	183 (40.5)	6 (37.5)	197 (40.7)
	A/G	69 (53.5)	175 (47.4)	56 (52.8)	189 (48)	24 (51.1)	221 (48.9)	10 (62.5)	236 (48.8)
	G/G	12 (9.3)	39 (10.6)	6 (5.7)	46 (11.7)	3 (6.4)	48 (10.6)	0 (0)	51 (10.5)

Supplementary Table S3. Frequencies of the most common haplotypes in preterm newborns, depending on incidence of respiratory distress syndrome, early-onset infection, pneumonia and sepsis. None of associations analysed apperaed significant (p>0.05).

Haplotype	Respiratory distress syndrome				Early-onset infection				Pneumonia				Sepsis			
	Yes		No		Yes		No		Yes		No		Yes		No	
	N	frequency	N	frequency	N	frequency	N	Frequency	N	frequency	N	frequency	N	frequency	N	frequency
AGAG	83	0.322	222	0.302	61	0.288	246	0.312	28	0.292	279	0.309	8	0.25	299	0.309
GGAA	62	0.24	178	0.242	51	0.241	192	0.244	23	0.24	220	0.243	10	0.313	233	0.241
AAAA	44	0.171	147	0.2	46	0.217	146	0.185	24	0.25	168	0.186	8	0.25	184	0.19
AGAA	23	0.089	76	0.103	21	0.099	78	0.099	11	0.115	88	0.097	3	0.094	96	0.099
GGCA	27	0.105	70	0.095	23	0.108	74	0.094	5	0.052	92	0.102	1	0.031	96	0.099

Supplementary Table S4. Frequencies of the most common diplotypes in preterm newborns, depending on incidence of respiratory distress syndrome, early-onset infection, pneumonia and sepsis. None of associations analysed apperaed significant (p>0.05).

Diplotype		Respiratory distress syndrome				Early-onset infection				Pneumonia				Sepsis			
		Yes		No		Yes		No		Yes		No		Yes		No	
		N	%	N	%	N	%	N	%	N	%	N	%	N	%	N	%
1	AGAG/GGAA	28	21.7	64	17.4	20	18.9	74	18.8	8	16.7	86	19	5	31.3	89	18.4
2	AGAG/AAAA	14	10.9	46	12.5	16	15.1	44	11.2	7	14.6	53	11.7	3	18.8	57	11.8
3	AAAA/GGAA	10	7.8	35	9.5	9	8.5	37	9.4	7	14.6	39	8.6	2	12.5	44	9.1
4	AGAG/AGAG	12	9.3	29	7.9	6	5.7	35	8.9	3	6.3	38	8.4	0	0	41	8.5
5	AGAG/GGCA	6	4.7	27	7.3	4	3.8	29	7.4	1	2.1	32	7.1	0	0	33	6.8
6	AAAA/GGCA	8	6.2	21	5.7	7	6.6	22	5.6	1	2.1	28	6.2	1	6.3	28	5.8
7	AGAA/GGAA	7	5.4	20	5.4	8	7.5	19	4.8	5	10.4	22	4.9	1	6.3	26	5.4
8	GGAA/GGAA	4	3.1	24	6.5	5	4.7	23	5.8	1	2.1	27	6	1	6.3	27	5.6
9	AGAA/AGAG	7	5.4	17	4.6	6	5.7	18	4.6	4	8.3	20	4.4	0	0	24	5
10	AAAA/GGAG	7	5.4	14	3.8	4	3.8	17	4.3	3	6.3	18	4	2	12.5	19	3.9

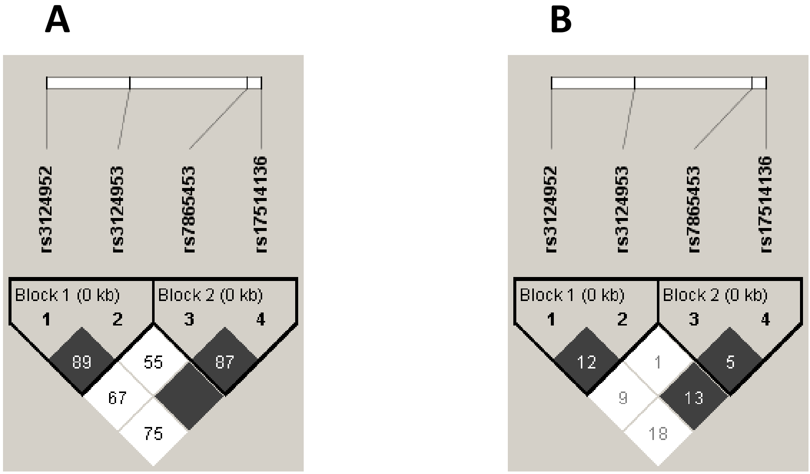

Supplementary Figure S1. Linkage disequilibrium analysis of promoter rs3124952 (-986 A>G), rs3124953 (-602 G>A), rs7865453 (-64 A>C), and rs17514136 (-4 A>G) *FCN2* single nucleotide polymorphisms. The numbers in the grid refer to D′ (**A**) and r^2 (**B**) parameters of the given pairs of SNPs. Bolded triangles shows haplotype blocks identified using four gamete rule test.

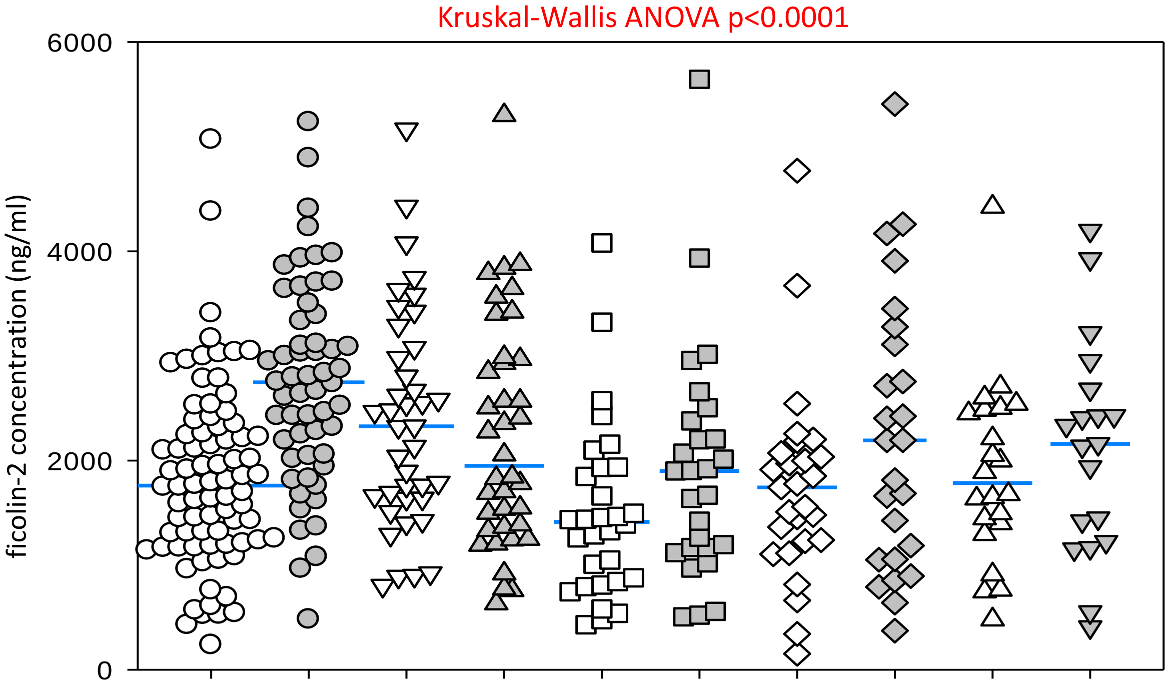

diplotype	1	2	3	4	5	6	7	8	9	10
**median ficolin-2**	**1761**	**2748**	**2327**	**1950**	**1415**	**1900**	**1743**	**2192**	**1785**	**2159**
**p (*vs.* 2)**	<0.000001		0,067	0,013	<0.000001	0.0002	0,00001	0.04	0.0003	0.02
**p (*vs.* 5)**	0.006	<0.000001	0,006	0,04		0.11	0,17	0.035	0.0507	0.034

Supplementary Figure S2. Individual concentrations of ficolin-2 in cord sera from preterm newborns, corresponding to 10 most common *FCN2* gene promoter diplotypes. Blue bars represent median values (given below the graph in bold). Medians related to diplotypes 2 (the highest) and 5 (the lowest one) were compared with remaining values using Mann-Whitney *U* test. Corresponding *p* values are given below the graph in red and blue, respectively.

Diplotypes: 1 – AGAG/GGAA; 2 – AGAG/AAAA; 3 – AAAA/GGAA; 4 – AGAG/AGAG; 5 – AGAG/GGCA; 6 – AAAA/GGCA; 7 – GGAA/GGAA; 8 – AGAA/GGAA; 9 – AGAA/AGAG; 10 – AAAA/GGAG

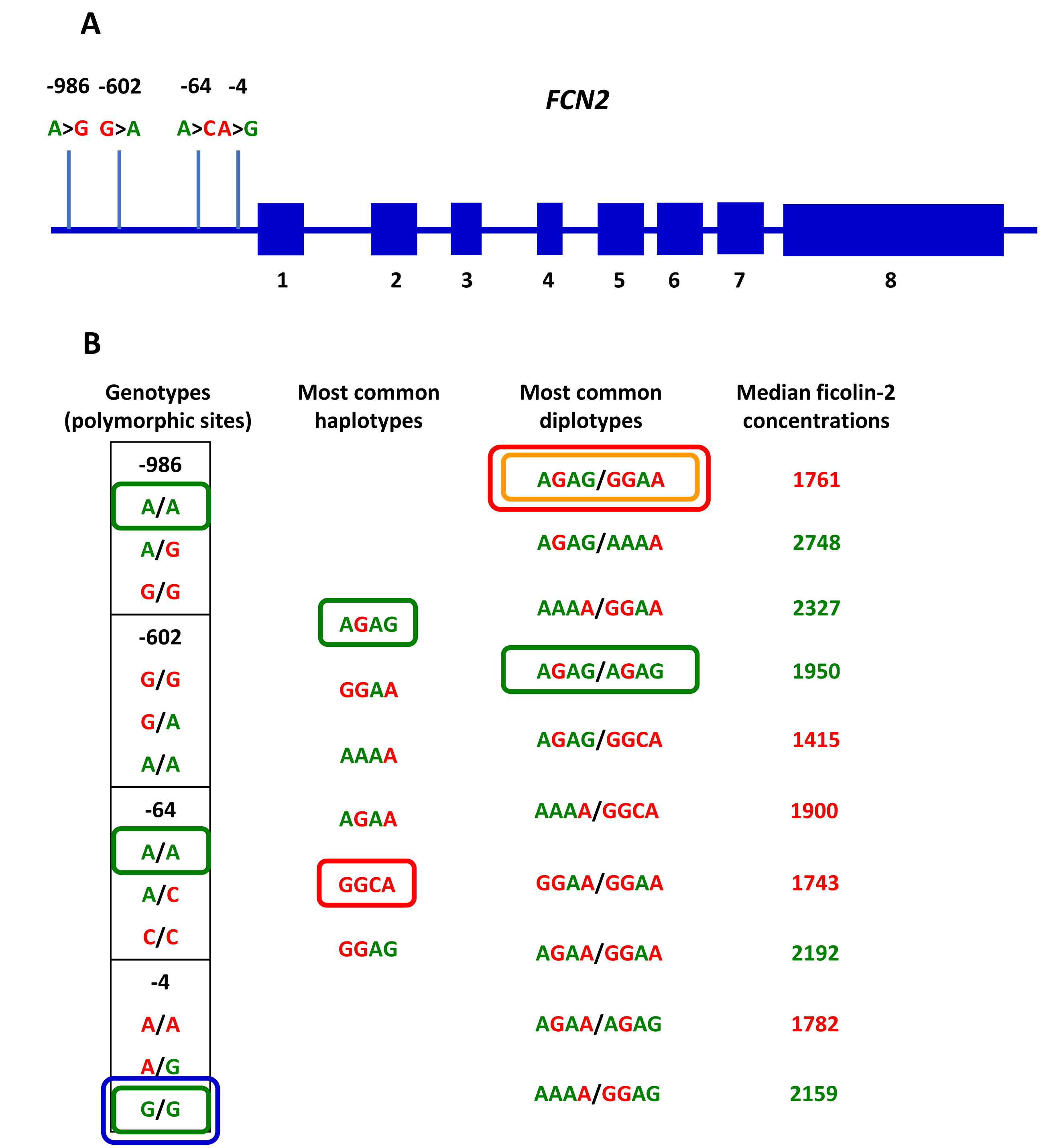

Supplementary Figure S3. A: Scheme of the *FCN2* gene with investigated promoter polymorphic sites. Alleles associated with higher gene expression are marked in green while those with lower – with red. Exons 1-8 are shown as blue rectangles. **B:** Genotypes corresponding to polymorphic sites, most common haplotypes, diplotypes and median ficolin-2 concentrations in cord sera (ng/ml), related to demonstrated promoter diplotypes. Alleles corresponding to particular sites, associated with higher gene expression are marked in green while those with lower – with red. Median ficolin-2 levels higher than median for the whole cohort are marked in green while those lower – in red. Genotypes: A/A (-986), A/A (-64), G/G (-4), all corresponding to relatively high *FCN2* gene expression; related AGAG haplotype and AGAG/AGAG diplotype were found associated with lower risk of very low birthweight (green boxes). Furthermore, G/G variant at -4 corresponds to lower risk of birth at gestational age <33 weeks (blue box). The GGCA haplotype (all alleles related to lower gene expression) was found associated with higher risk of VLBW (red box) while AGAG/GGAA diplotype with higher risk of both VLBW and GA <33 weeks (red and orange boxes).
